# The role of change in fertility desire on change in family planning use: A longitudinal investigation in urban Uttar Pradesh, India

**DOI:** 10.12688/gatesopenres.12956.2

**Published:** 2019-06-18

**Authors:** Ujjaval Srivastava, Anjali Singh, Prashant Verma, Kaushalendra Kumar Singh

**Affiliations:** 1 Department of Statistics, Banaras Hindu University, Varanasi, Uttar Pradesh, 221005, India; 2 Indian Statistical Service, National Statistical System Training Academy, Greater Noida, 201310, India; 3 Analytics Department, Global IT Centre, State Bank of India, Navi Mumbai, Maharashtra, 400614, India

**Keywords:** Fertility desire, contraceptive use, Urban Health Initiative, Measurement Learning & Evaluation, causal change.

## Abstract

**Background: **Reproductive choice is one of the rights of any woman, but women are often ambivalent towards fertility desires and choice of contraception. Our study explores how the change in fertility desires influence the change in use of modern contraception over time in six cities of Uttar Pradesh, India.

**Methods** **:** Data for this study comes from the Measurement, Learning and Evaluation (MLE) Project for Urban Health Initiative in six cities of Uttar Pradesh. Our study sample consists of 8735 women (weighted n=8655) who were fertile, non-sterilized and non-pregnant at the time of baseline survey. Potential bias due to lost to follow up was addressed using inverse probability weighing and then generalized estimating equations were applied to get odds for change in use of modern contraceptives.

**Results:** Contraceptive use increased by different magnitudes from baseline to endline across all six cities. At baseline and endline, women who desired no more children reported a higher use of modern contraception than those who desired more children over time. Women from all cities who desired no more children at baseline had higher odds of modern contraceptive use than that of women who desired more children. The tempo of change in use of modern contraception over time among women with different fertility desires differed across the considered cities.

**Conclusion** **:** Although there were city-wise differences observed, women’s fertility intentions have an impact on their use of modern contraceptives over the time period between baseline to endline. To obtain greater insight into city-level differences, mixed method studies will be more effective.

## Introduction

Women’s fertility desire is one of the most widely used measure for assessing fertility preferences. To measure women’s fertility intentions, we usually ask “ *would you like to have a/another child, or would you prefer not to have any more children?*” Several studies both at individual and couple level have shown that women’s fertility intentions can be a powerful predictor of women’s contraceptive behaviour and fertility outcomes ^1– 9^ and it has substantial policy implications in framing future family planning (FP) strategies in all countries.

The importance of knowing women’s fertility preferences is that it gives us idea about proportion of women who want to delay or limit childbearing. This knowledge helps providers to assist women in choosing most suitable protective device to control fertility or none at all. Another need to understand the fluidity in the fertility intentions and contraceptive use is assessing the level of unmet need and demand of contraceptives and estimating the extent of unintended and mistimed pregnancies.

Reproductive choice is one of the most fundamental rights of a couple, and therefore couples should be free to reproduce as well as use contraceptives during their reproductive span. Empowering a woman to control her fertility allows her to complete her education or employment aspirations. Evidence from both developing ^1– 3, 5, 10, 11^ and developed countries ^12– 14^ shows that fertility desires of women frequently vary with corresponding contraceptive use, and women are often uncertain about getting pregnant and choosing a suitable contraceptive among several available contraceptive methods. Previous research revealed that questions about whether or not a woman want to have another child rarely account for the variety of contradictory emotions that women often experience regarding pregnancy and childbirth ^3, 15– 17^, or the role of external influence on such decisions, including the husband’s desires, expectations of other family members and community, and religious and social norms ^18– 21^. Another study in Ghana ^22^ revealed that women’s attitude towards use of modern contraceptives is affected by the education status of women and also by husband’s attitude. A study in Nigeria ^23^ suggested that parity is an important predictor for the relationship between a women’s desired fertility status and actual behavior, including contraceptive behavior. It also identified that the desire of women and her husband plays a key role in predicting the fertility behavior of couple. A study in Madhya Pradesh ^7^ found that fertility desire and contraceptive behavior of women aged under 30 or less were more likely to be inconsistent with their previously stated fertility desire and use than older women and concluded that women with no more desire for another child and who intended to use modern contraceptive method were more likely to use a contraceptive than their counterpart. Some studies ^24, 25^ suggested that there is a direct linkage between preference for a son and fertility intentions.

According to most recent National Family Health Survey ^26^, modern contraceptive use among currently married women aged 15–49 years in all six cities assessed (Agra, Aligarh, Allahabad, Gorakhpur, Moradabad, Varanasi) lie between 32.7% (Allahabad) and 42.6% (Varanasi), but we found wide variation in unmet need for FP between eastern regions (Allahabad (23.1%), Gorakhpur (23.3%), Varanasi (16.4%)) and western regions (Agra (10.2%), Aligarh (12.7), Moradabad (10.4)). Measuring unmet need is not enough for policy makers and FP service providers, the knowledge of whether women with equivocal feelings about their future fertility intentions have unmet need for FP may help them in taking better decisions regarding policy formulations. A similar type of study in Sub-Saharan Africa ^27^ attempted to study the relationship between fertility motivations and modern contraceptive use over time in high-fertility locations (Ghana, Nigeria and Ethiopia) through panel data. The present study is an attempt to explore the relationship between women’s fertility desires and their contraceptive behavior through causal inference in urban settings of Uttar Pradesh, India.

## Research hypotheses

Our study aims to investigate for following research questions, which are converted into testable hypotheses:

1) Our first hypothesis is that whether the women are differing in their fertility intentions and also differ in their contraceptive use?, i.e., if fertility intentions are powerful predictor of FP use then women who do not desire more children at baseline should report higher use of contraceptives both at baseline and endline in contrast to women who desire more children at baseline.
2) Whether the change observed in use of modern contraceptives between baseline to endline was same for women with different fertility desires?, i.e., our hypothesis is that women who desire more children at baseline would state lower use of modern contraceptives at endline and women who do not desire more children at baseline would report higher use of modern contraceptives at endline.
3) Our last hypothesis is that the relationship between women’s fertility desires and use of modern contraceptives over time would remain same after adjusting for potential confounding variables (Baseline education, parity, wealth, caste, religion, age, residence and having son at home).

## Methods

### Urban Reproductive Health Initiative (URHI)

The data source for this study is a multi-city longitudinal study from the Measurement, Learning & Evaluation (MLE) Project led by Bill & Melinda Gates Foundation for the evaluation of URHI in Uttar Pradesh, India ^28, 29^. It is implemented with an aim to reduce unintended pregnancy, infant and maternal mortality by increasing desire to use FP services and access to family FP services among the urban poor. The URHI program implemented several demand and supply side interventions. The demand side interventions include mass-media campaigns to promote FP services, peer outreach and community mobilization in which a community health worker (CHW) visits every house of a slum and informs individuals about FP services, counsels them and provides short-term methods of FP if they required. On the other side, supply interventions includes integration of FP with post-abortion and postpartum services, involvement of the private sector in FP services and training of health care providers on technical and counselling skills to promote FP services.

### Sampling design

The longitudinal study consists of baseline, midline and endline surveys, each at intervals of 2 years; the protocol has been described in full previously ^28, 29^. MLE uses quasi-experimental design to collect cross-sectional and longitudinal data at individual level from men and women and service delivery point data. By interviewing same women at multiple times we can examine how women’s exposure to the URHI program will affect the changes in their subsequent contraceptive behaviors, adjusting for their baseline potential confounding variables. Baseline survey data was collected from a representative sample of 17,643 married women from the urban slums and non-slums of six cities (Agra, Aligarh, Allahabad, Gorakhpur, Moradabad and Varanasi) of Uttar Pradesh, India in early 2010. Geographic information system (GIS) maps were used to develop sampling frame, and a multi-stage sampling technique was used for sample selection. Each city is divided into set of sampling units of slum and non-slum in order to achieve equal size sample from slum and non-slum population. At first stage a sample of 64 primary sample units (PSU) were selected and at next stage, a sample of 30 households were selected from each selected PSUs from both slum and non-slum areas. Each household and women of baseline survey who was still residing in one of the six cities was followed and an attempt to re-interview each was made at mid-term and endline survey. For the data collection at endline survey in 2014, baseline questionnaires were modified to some extent and 14,043 women were successfully interviewed at endline, with a response rate of 83.6%. Weights were used to adjust oversampling of slums and selection bias due to lost to follow up in longitudinal study. At endline, approximately 77% of the baseline cohort of Agra were successfully interviewed. In Aligarh & Moradabad (82% of baseline cohort) and Allahabad, Gorakhpur & Varanasi (79% of baseline cohort) were successfully interviewed. Mostly women who were lost at endline moved away from the study cities. Additionally, some women refused to participate and some were died during the 5-year period. For more details of complete protocols of MLE intervention one can refer to the baseline report ^28, 29^.

Our study sample is limited to women who were fertile and non-sterilized and non-pregnant at baseline survey. We have not used midline survey data, as the midline survey was carried in Agra, Aligarh, Allahabad and Gorakhpur only, and here we want to see change in modern contraceptives between baseline to endline in all six cities.

Women’s fecund status is assessed by their response on hysterectomy and menopause and “ *can’t get pregnant*”. Sterilization included women’s sterilization or her husband’s sterilization. We also excluded women who were pregnant at baseline survey since their future fertility desires depended on the outcome of current pregnancy. Thus, the final analysis sample consists of 8735 women (weighted n=8655). Around 19% of women who were found eligible at baseline were ineligible to provide response to fertility desire (i.e. either they were not able to have children due to menopause/hysterectomy or they were sterilized at time of endline). Women were asked regarding fertility intentions and modern contraceptive use at both baseline and endline surveys. The primary outcome was change in the use of modern contraceptives observed over the two-survey point. Women were asked “ *would you like to have a/another child?*” ** at baseline and endline. Their response were categorized into “ *desire more*”, “ *desire no more*” and “ *undecided*” *.* A total of 21 eligible women at baseline and 26 women at endline who were undecided about their fertility desires and 4 women who had missing information at baseline were grouped into the “ *desire more*” category, as per previously described convention ^30^. Women were classified as modern contraceptive users if the woman or her husband was using one of the modern FP methods (oral contraceptive pills, intrauterine devices (IUDs), male/female condom, diaphragms, lactational amenorrhea method (LAM), implants, emergency contraception, and injectables). Baseline education, parity, wealth, caste, religion, age, residence and having a son at home were variables used as potential confounders. Here education was categorized as “No education”, “Primary”, “Secondary” and “Higher than secondary”. Women’s parity (the number of children ever born) was grouped into 0-1, 2-4 and 5+. A wealth index was created based on data from a list of consumer durables using principal component analysis as per the definition given by Demographic Health Surveys. Wealth index divided the whole population into five equal-sized groups (poorest, poor, medium, rich & richest). Caste was classified into scheduled caste (SC)/scheduled tribe (ST), other backward class and general caste. The castes which were the elites of ancient Indian society are classified as general and rest are classified in lower castes. Lower castes are classified into 3 categories: SC, who were ‘untouchables’ (dalit) in ancient India; ST, who did not accept the caste system and preferred to live in forests and mountains; and other backward caste, the remaining lower castes. In our study, ST was combined with SC, since ST constitutes less than 1% of the total sample. Religion was grouped into Muslim and non-Muslim categories. Here non-Muslim group contains Hindu & other religions. Women’s ages were categorized as <25 years, 25–34 years and 35+ years. Residence was dichotomized into slum and non-slum. Presence or absence of son at baseline survey was also used as a confounding variable.

### Statistical analysis

We have used panel data to estimate the influence of change in fertility desire on change in modern contraceptive use over time among women who desired more children relative to women who desired no more children at baseline. Descriptive analysis was carried out and using chi-square analysis, selected categorical variables were cross-tabulated with modern contraceptive use, with *p*<0.001, *p*<0.01 and *p*<0.05 indicating a statistically significant difference. Significant variables were kept as confounding variables in the final model. Thereafter, we have used propensity score (PS) methods. PS is an equalizing score that tells us that women who desired no more and who desired more children at baseline with matching PS will have similar distribution of observed baseline covariates (education, parity, wealth, caste, religion, age, residence and having son at home).

Inverse probability weighing (IPW) is as method used to obtain PS and for adjusting selection bias introduced because of loss to follow up. In IPW, we created pseudo-population by means of re-evaluating the contribution of each woman who was not lost to follow up for a given set of baseline covariates ^31^. To construct a denominator of IPW for women who desired no more children at baseline, we estimated the conditional probability of being followed up for a given set of baseline covariates. For numerator of IPW, the probability of being followed up with only intercept and no covariates was estimated. Similarly, IPW for women who desired more children at baseline was calculated. Next using these IPWs as weights, we used generalized estimating equations (GEE) to fit a generalized linear model (GLM) with binomial family, logit link function and exchangeable correlation structure to produce unbiased estimates of difference in modern contraceptive use over time and 95% confidence intervals (CI) were also obtained using robust standard errors (SE). We want to estimate

*logit* Pr ( *Y _it_* = 1) = *β*_0_ + *β*_1_ *X _it_* + *β*_2_ *Treatment _it_* + *β*_3_ *Time _t_* + *β*_4_ *Treatment _it_* × *Time _it_* for *t* = 0, 1; *i* = 1,2,… *N*.

Here, *Y _it_* is binary outcome variable for *i* = 1,2,… *N* observations in the sample at time *t* = 0 (2010) and = 1 (2014), i.e., *Y _it_* = 1 if women is currently using modern contraceptives and *Y _it_* = 0 otherwise. *X _it_* represents a vector of confounding covariates (education, parity, wealth, caste, religion, age, residence and having son at home). *Treatment _it_* is a dummy variable i.e. *Treatment _it_* = 1 if women desired no more children at baseline and *Treatment _it_* = 0 otherwise. *Time _t_* is also a dummy variable, which takes value 1 at endline and 0 at baseline. *e* ^*β*_2_^ gives the odds ratio (OR) of modern contraceptive use among women who desired no more children relative to women who desired no more children at baseline. Likewise *e* ^*β*_3_^ provides the OR of modern contraceptive use at endline with respect to baseline among women who desired more children at baseline. Further *e* ^*β*_4_^ yields the OR comparing causal change in modern contraceptive use over time among women who desired no more children in contrast to women who desired more children at baseline.

## Results

### Use of modern contraception

Table 1 shows the city-wise percentage distribution of women using modern contraceptive methods at baseline and endline. In addition to this, it also shows the proportion of women who became ineligible (due to menopause, hysterectomy or sterilization) to provide answer for their fertility desire at endline. The magnitude of change in use of modern contraceptives between baseline and endline differed across cities, but the proportion of women using modern contraceptives increased over all six cities (Agra (0.5%), Aligarh (6.5%), Allahabad (6.3%), Gorakhpur (5%), Moradabad (3.6%) and Varanasi (4.3%)) of Uttar Pradesh. However, this increase is fictitious as 5.7% (Agra), 4.7% (Aligarh), 6.1% (Allahabad), 4.4% (Gorakhpur), 6.8% (Moradabad) and 7.7% (Varanasi) of modern contraceptive users at baseline became infecund at endline, and new users replaced them at endline among the women who were using modern contraceptive at the base period. Therefore, the real increase would be more than that seen in the column of the Table 1, represented by the heading ‘endline’.

**Table 1.** City-wise percentage distribution of women using modern contraceptive methods.

City	Weighted sample size (N)	Current use of modern contraceptives [%]		Percentage share of baseline women who become ineligible to provide answer for fertility desire at endline	Percentage of baseline modern contraceptive users who become infecund at endline
		Baseline	Endline		
Agra	1439	40.1	40.6	17.1	5.7
Aligarh	1758	36.7	43.2	15	4.7
Allahabad	1309	36.8	43.1	21.2	6.1
Gorakhpur	1305	34	39	17.1	4.4
Moradabad	1415	46.7	50.3	18.2	6.8
Varanasi	1429	40.8	45.1	21.2	7.7
Overall	8655	39.2	43.6	18.1	5.9

### Use of modern contraception by background characteristics

Table 2 shows the city-wise distribution of women using modern contraceptives according to their background characteristics. The percentage of modern contraceptive users amongst women who desire more children at baseline decreased over time in all cities except Aligarh. The magnitude of this decrement was highest for Gorakhpur (7.1%) and lowest for Varanasi (0.4%). The above proportion for the women, who did not desire for more children at baseline, increased in all the six cities. Analyzing age-group-wise variation in percentage of modern contraceptive users from baseline to endline, it has been observed that for the age groups <25 and 25–35 years, use of modern contraception increased, while for the higher age group (>35 years), proportion of modern contraceptive users decreased. For all six cities, the proportion of modern contraceptive users was stratified according to their level of education. The greatest proportion were in the highest education category (higher than secondary) at both baseline and endline. The major increase in modern contraceptive users from baseline to endline across all the cities is attributed to those women who had either no education or education up to secondary level. Apart from this, the largest decrease in modern contraceptive over time was found among highly educated women. Across the cities, the proportion of modern contraceptive users among each caste (SC/ST, Other backward, General) increased over time except for the general caste group at Agra (3.9% decrease), Gorakhpur (0.2% decrease) and Moradabad (2.7% decrease). Among Muslims and non-Muslims, the share of modern contraceptive users increased over the follow-up period, except for Muslims in Gorakhpur. When we examined the amount of modern contraceptive use among slum and non-slum samples, more users were found in the non-slum sample than in the slum sample. After comparing modern contraceptive use across all cities according to wealth quintile, women in the poorest quintile had lower use of modern contraceptives. However, women in the richest quintile, both at baseline and endline, made up a greater proportion of modern family planning users than other groups. Notably, for women with parity below 1 and 2-4, the percentage of modern contraceptive users increased over time; however, the highest increase was found for the cities Aligarh and Varanasi (both 14.1%), but for women with a parity of greater than five, no regular increase or decrease could be seen. The share of modern family planning users had increased over time among women with at least one son and without a son. The amount of increase in modern contraceptive users among women who had at least one son was found highest at Aligarh (15.3%) and lowest at Moradabad (0.5%).

**Table 2.** City wise percentage distribution of women using modern contraceptive methods according to selected background characteristics.

Variable	Modern contraceptive users											
	Agra (weighted N=1439)		Aligarh (weighted N=1758)		Allahabad (weighted N=1309)		Gorakhpur (Weighted N=1305)		Moradabad (Weighted N=1415)		Varanasi (Weighted N=1429)	
	Baseline (%)	Endline (%)	Baseline (%)	Endline (%)	Baseline (%)	Endline (%)	Baseline (%)	Endline (%)	Baseline (%)	Endline (%)	Baseline (%)	Endline (%)
**Fertility desire**												
Desire more children	28.5 ^c^	24.5 ^c^	22.7 ^c^	27.3 ^c^	24.5 ^c^	21.8 ^c^	27.5 ^c^	20.4 ^c^	30.6 ^c^	24.3 ^c^	27.7 ^c^	27.3 ^c^
Desire no more children	46.8	48.2	42.2	51.2	43.2	51.0	37.3	43.0	54.9	62.1	47.5	50.6
**Age-group**												
<25	30.6	37.2	23.3	46.3	29.3	43.8	27.0	38.1	32.1	52.9	28.0	44.1
25–34	47.7	51.7 ^c^	42.7 ^c^	53.5 ^a^	43.4 ^c^	54.0 ^b^	38.9 ^c^	48.8 ^b^	52.5 ^c^	62.5 ^b^	47.8 ^c^	56.6 ^c^
35+	35.4 ^c^	26.4 ^b^	36.7 ^c^	28.1 ^c^	31.2	25.6 ^c^	31.9	25.5 ^c^	47.7 ^c^	32.9 ^c^	37.8 ^b^	27.6 ^c^
**Education**												
No education	27.8	32.6	25.2	34	17.7	32.5	17.3	28.8	37.0	42.5	28.3	28.6
Primary	38.5 ^a^	43.4 ^a^	33.3 ^a^	39.6	29.8 ^b^	38.6	31.7 ^c^	33.8	46.9 ^a^	58.5 ^b^	40.1 ^b^	39.6 ^b^
Secondary	46.3 ^c^	43.4 ^c^	42.9 ^c^	50.5 ^c^	37.1 ^c^	43.1 ^b^	37.5 ^c^	42.9 ^c^	51.5 ^c^	57.5 ^c^	44.1 ^c^	52.6 ^c^
Higher than secondary	55.5 ^c^	50.6 ^c^	56.3 ^c^	55.3 ^c^	50.1 ^c^	50.9 ^c^	48.9 ^c^	47.1 ^c^	58.6 ^c^	50.5 ^a^	55.7 ^c^	61.2 ^c^
**Caste**												
SC\ST *	34.2	34.4	28.9	32.6	24.5	37.5	26.0	31.6	41.2	44.9	34.6	38.3
Other backward caste	39.4	44.0 ^b^	33.4	45.3 ^c^	36.2 ^b^	43.8	33.0 ^a^	41.3 ^b^	43.2	50.9	40.1	44.1
General caste	46.5 ^c^	42.6 ^a^	45.2 ^c^	47.6 ^c^	42.0 ^c^	44.1	39.8 ^c^	39.6 ^a^	53.8 ^b^	51.1	45.2 ^b^	50.7
**Religion**												
Non-Muslims **	39.8	53.3	36.1	47.3	38.3	58.4	32.3	57.7	46.0	60.8	43.5	64.0
Muslims	40.8	51.4 ^b^	37.9	47.5	32.4 ^c^	44.0 ^a^	40.5 ^b^	39.6	47.6 ^c^	50.7	34.0 ^c^	40.7 ^c^
**Residence**												
Non-Slum	42.5	40.3	38.4	40.9	41.5	45.6	38.2	39.8	50.8	51.5	46.0	44.0
Slum	37.8	40.9	35.2	45.4	30.8 ^c^	40.0 ^a^	29.6 ^b^	38.3	42.3 ^b^	49.1	36.2 ^c^	46.1
**Wealth**												
Poorest	26.8	31.3	22.2	34.9	21.0	43.3	18.6	31.2	32.0	46.7	25.9	32.7
Poor	31.6	39.0	29.1 ^a^	39.6	30.4 ^a^	41.7	31.2 ^c^	36.5	39.6	52.2	34.0 ^a^	37.6
Medium	38.6 ^b^	38.3	34.0 ^b^	46.8 ^b^	34.2 ^b^	33.6 ^a^	41.0 ^c^	40.6 ^a^	48.5 ^c^	47.8	42.7 ^c^	46.0 ^b^
Rich	49.2 ^c^	45.6 ^b^	41.7 ^c^	43.7 ^a^	44.7 ^c^	47.2	38.0 ^c^	42.6 ^b^	54.9 ^c^	51.0	47.2 ^c^	50.8 ^c^
Richest	50.7 ^c^	46.7 ^c^	54.3 ^c^	49.4 ^c^	49.1 ^c^	48.7	48.0 ^c^	48.0 ^c^	58.8 ^c^	54.1	53.7 ^c^	58.1 ^c^
**Parity**												
0–1	30.8	35.0	24.3	38.4	28.6	37.3	28.0	31.4	30.4	41.2	27.7	41.8
2–4	47.8 ^c^	46.7 ^c^	43.5 ^c^	50.2 ^c^	43.8 ^c^	47.3 ^b^	39.0 ^c^	46.0 ^c^	55.5 ^c^	58.2 ^c^	50.7 ^c^	52.9 ^c^
5+	29.3	29.6	31.9 ^b^	31.5 ^a^	21.7	36.4	24.7	24.7	44.5 ^c^	41.0	31.4	27.1 ^c^
**Having son at home**												
Yes	35.5	39.0	35.2	50.5	39.3	46.8	34.0	36.0	43.3	43.8	43.7	44.9
No	40.9	40.9	36.9	42.2 ^a^	36.1	42.1	34.0	39.7	47.3	51.5 ^b^	40.2	45.2

^a^ p<0.05, ^b^ p<0.01, ^c^ p<0.001. *Frequency of ST constitutes less than 1% and therefore grouped with SC. **Non-Muslim constitutes Hindu and other religions. ST, scheduled tribe; SC, scheduled caste.

Table 3 and Table 4 depicts the change in fertility desire and use of modern contraceptives between baseline and endline among eligible women with complete data. In Table 3, the magnitude of change in fertility desire was comparable across six cities. Approximately 3–5% of women who desired no more children at baseline reported that they desired more children at endline. However, more than half of the women (49–57%) who desired more children at baseline reported that they desired no more children at endline. As shown in Table 4, approximately 57%, 63%, 65%, 59%, 66% and 64% of the users in Agra, Aligarh, Allahabad, Gorakhpur, Moradabad and Varanasi, respectively, are also using contraception at endline survey. The shift from non-users at baseline to users at endline was highest in Moradabad (37%) followed by Varanasi (32%), Aligarh (32%), Allahabad (31%) and Agra (30%), and was lowest in Gorakhpur (29%). Change in modern contraceptive use in opposite direction from use at baseline to non-use at endline was slightly lower in Moradabad (35%) but higher across rest five cities with a range of 36% (Allahabad) to 43% (Agra).

**Table 3.** City-specific change in fertility desires between baseline to endline.

Baseline desire for another child	Endline desire for another child, %											
	Agra		Aligarh		Allahabad		Gorakhpur		Moradabad		Varanasi	
	No more	More	No more	More	No more	More	No more	More	No more	More	No more	More
No more	95.8	4.2	96.2	3.8	95.1	4.9	95.5	4.5	96.9	3.1	95.3	4.7
More	57.2	42.8	54.3	45.7	52.5	47.5	49.3	50.7	57.0	43.0	56.0	44.0

**Table 4.** City-specific change in use of modern contraception between baseline to endline.

Baseline use of modern contraception	Endline use of modern contraception, %											
	Agra		Aligarh		Allahabad		Gorakhpur		Moradabad		Varanasi	
	Non users	Users	Non users	Users	Non users	Users	Non users	Users	Non users	Users	Non users	Users
Non users	70.3	29.7	68.0	32.0	69.4	30.6	70.9	29.1	62.8	37.2	67.8	32.2
Users	43.1	56.9	37.4	62.6	35.5	64.5	41.7	58.3	34.7	65.3	36.1	63.9

**Figure 1.**
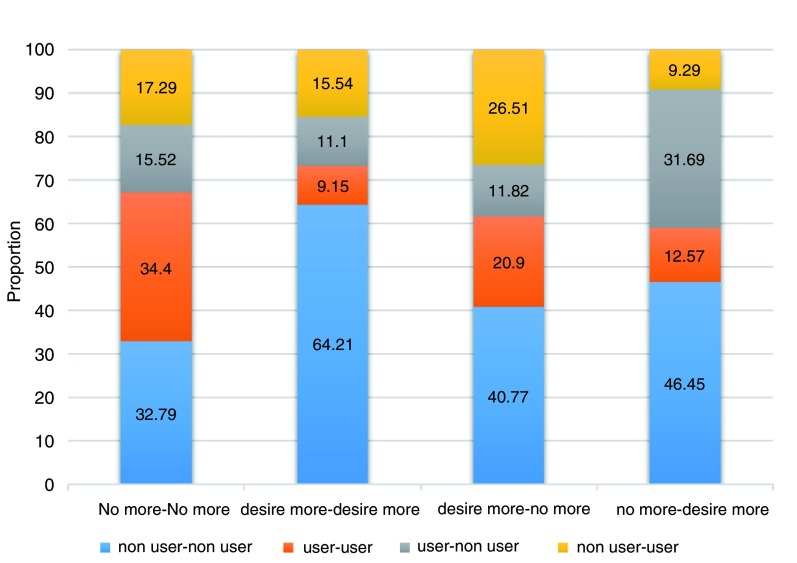
Changing status of modern contraceptive use over time according to change in fertility desires.

Figure 1 shows the distribution of change in contraceptive users according to change in their fertility desires. Of the women who have an unchanged desire for no more children from baseline to endline, around 34% were coherent users of modern contraceptives, followed by 33% who were consistent non-users and 33% who changed their status of modern contraceptive user. Among women who desired more children both at baseline and endline, around 64% were consistently not using any modern contraceptives, 9% were regular users and the remaining number were inconsistent in using contraception. Around 27% of women whose fertility desire changed from desiring more children at baseline to no more children at endline swapped from non-users to users of modern contraceptives, while 41% and 21% of women were consistently not using and using contraception respectively. In addition to above, nearly 32% women whose fertility motivations have changed from “desire no more children” at baseline to “desire more children” at endline, were shifted from users to non-users of modern contraceptives, while 46% of total women who belong to the above category persistently did not use any modern contraception. Furthermore, 13% of women whose fertility desires have shifted from “desire no more” to “desire more”, were consistently using modern methods of contraception. It is clear from above figure that inconsistency in using modern contraception was higher among women with ambivalent fertility desires.

Table 5 presents the results of multivariate GEE analysis. Table 5 presents findings from two models for each of the six urban cities of Uttar Pradesh. The first model is unadjusted for confounders and includes the key independent variable fertility desires, the time variable and an interaction between the two. The second model is adjusted for the potential confounders, which are age of women, educational status, caste, religion, residence (slum/non-slum), wealth status, parity and having son at home. Women from all cities who desired no more children at baseline had higher odds of being modern contraceptive users than women who desired more children. All adjusted and unadjusted ORs for the women who desired no more children at baseline relative to women who desired more children are significant. All women who desired no more children at baseline survey were reported higher contraceptive use at baseline survey than that of women having more desire of children. The adjusted OR for modern contraceptive use at endline with respect to baseline among women who desired more children reported lower use of modern contraceptives at all cities (0.43, 0.87, 0.54, 0.43 and 0.68 for Agra, Allahabad, Gorakhpur, Moradabad and Varanasi respectively) except Aligarh (1.60), but reached statistical significance only in Gorakhpur and Moradabad. The OR for the interaction term (Fertility desire × Time) shows the average effect of change in fertility desire between baseline and endline on change in modern contraceptive use over time. In other words, it shows the OR for change in modern contraceptive use over the follow up period among women who desired no more children at baseline with respect to those women who desired more children. The adjusted OR for the interaction term attained significance in Agra, Allahabad, Gorakhpur, Moradabad and Varanasi, with ORs of 1.78 (95% CI 1.40-2.53), 1.46 (95% CI 1.13-3.09), 2.19 (95% CI 1.24-3.84), 2.79 (95% CI 1.33-5.85) and 1.48 (95% CI 1.29-2.77), respectively. Additionally, the OR of (Time + Fertility desire × Time) indicates the OR for modern contraceptive use at endline with respect to baseline among women who desired no more children. All these adjusted ORs were in the hypothesized direction at all cities (1.74, 1.24, 1.55, 1.43, and 1.19 for Aligarh, Allahabad, Gorakhpur, Moradabad and Varanasi, respectively) except Agra (0.97), but did not attain statistical significance in Agra and Varanasi. This result means that, except for Agra, women who desired no more children at baseline survey were reported higher use of modern contraceptives at the time of endline survey in comparison to women having more desire of children at baseline. It is clear from the significance of overall regression that the changes observed in modern contraceptive use between baseline and endline for women who desired more children and those who desired no more children was not similar.

**Table 5.** City-specific unadjusted and adjusted odd ratios and 95% confidence intervals illustrating effect of change in fertility desires on change in use of modern contraception.

Covariate	Agra		Aligarh		Allahabad		Gorakhpur		Moradabad		Varanasi	
	OR (unadj.)	OR (adj.) ^d^	OR (unadj.)	OR (adj.) ^d^	OR (unadj.)	OR (adj.) ^d^	OR (unadj.)	OR (adj.) ^d^	OR (unadj.)	OR (adj.) ^d^	OR (unadj.)	OR (adj.) ^d^
**Fertility desire** **(Ref=Desire** **more)**	3.11 (2.21-4.39) ^c^	3.33 (2.28-4.87) ^c^	2.84 (1.98-4.08) ^c^	3.47 (2.41-5.00) ^c^	2.45 (1.58-3.78) ^c^	2.70 (1.69-4.30) ^c^	1.60 (1.06-2.41) ^a^	1.44 (0.92-2.27)	2.37 (1.58-3.53) ^c^	2.30 (1.44-3.69) ^c^	1.76 (1.16-2.68) ^c^	1.82 (1.19-2.77) ^b^
**Time** **(Ref=Baseline)**	1.31 (0.70-2.43)	0.98 (0.50-1.90)	1.47 (0.84-2.57)	1.60 (0.90-2.84)	0.98 (0.56-1.70)	0.87 (0.51-1.50)	0.63 (0.38-1.04)	0.54 (0.32-0.92) ^a^	0.45 (0.23-0.87) ^a^	0.43 (0.22-0.86) ^a^	0.66 (0.37-1.17)	0.68 (0.39-1.19)
**Fertility** Desire*Time	1.58 (1.29-2.15) ^a^	1.78 (1.40-2.53) ^b^	0.93 (0.51-1.69)	0.83 (0.45-1.53)	1.60 (0.76-2.81)	1.46 (1.13-3.09) ^b^	1.82 (1.09-3.35) ^a^	2.19 (1.24-3.84) ^b^	2.71 (1.31-5.57) ^b^	2.79 (1.33-5.85) ^b^	1.66 (1.18-3.14) ^b^	1.48 (1.29-2.77) ^b^
**Time + Fertility** Desire*Time	1.18 (0.65-1.95)	0.97 (0.61-1.96)	1.82 (1.12-2.09) ^b^	1.74 (1.04-2.15) ^a^	1.31 (1.09-1.46) ^a^	1.24 (0.97-1.46)	1.63 (1.18-2.04) ^a^	1.55 (1.26-1.92) ^a^	1.45 (1.23-1.87) ^b^	1.43 (1.21-1.86) ^a^	1.26 (0.37-1.67)	1.19 (0.37-1.54)
**constant**	0.29 (0.21-0.40) ^c^	0.07 (0.04-0.14) ^c^	0.26 (0.18-0.36) ^c^	0.06 (0.03-0.12) ^c^	0.30 (0.21-0.44) ^c^	0.04 (0.01-0.13) ^c^	0.34 (0.24-0.50) ^c^	0.04 (0.02-0.10) ^c^	0.50 (0.34-0.73) ^c^	0.10 (0.04-0.23) ^c^	0.49 (0.33-0.72) ^c^	0.02 (0.003-0.12) ^c^
**Observations** **in Panel data**	2558	2558	3167	3167	2185	2185	2491	2491	2636	2636	2487	2487
**p-value**	0.00	0.00	0.00	0.00	0.00	0.00	0.00	0.00	0.00	0.00	0.00	0.00

^a^ p<0.05, ^b^ p<0.01, ^c^ p<0.001, ^d^ models were adjusted for education, parity, wealth, caste, religion, age, residence and having son at home

## Discussion

We observed the highest increase in modern contraceptive use over time (6.5%) and the lowest proportion of women with inconsistent fertility desires (18.8%) in Aligarh. In Agra, although the level of increase in modern contraceptive use between baseline to endline was lowest (0.5%), women reported highest level of inconsistent fertility desires (24.2%). Our results suggest that changes in women’s fertility desire is strongly associated with changes in modern contraceptive use over time. Women who had consistent fertility desires were more likely to be consistent in their contraceptive use. Our findings reinforce the findings of other longitudinal and cross-sectional studies ^7, 10, 11, 14, 27, 32, 33^. However, our study is distinct from the previous ones as our study has additionally included caste, religion, residential status and presence of son at home as confounding variables. The significance of our results in urban settings indicates that if ambivalent feelings towards fertility desires and contraceptive use are prevalent in considerable amounts in urban areas (where literacy levels are comparatively higher than rural areas and where access to family planning centers is easy compared to that in rural areas), it is unclear what the situation of ambivalent women will be in the rural areas of India where there are several physical and socio-cultural barriers to use family planning methods. Nevertheless, this hypothesis should be tested further for several other cities as well as for rural areas. As we have speculated, women who desired no more children reported higher use of modern contraception relative to those women who desired for more children at both baseline and endline across all cities. The reasons for fluctuation in fertility intentions and modern contraceptive use are beyond the scope of analysis and requires more investigation. Earlier research ^14, 32, 34, 35^ illustrated that there may be various barriers for women taking the decision to use contraception, including husband’s desire, gender and social norms, problems to access and misconceptions about side effects of contraception. Another study in Michigan ^33^ revealed that only strong motivation to avoid being pregnant pushed women to use any form of contraception ^33, 35^.

In the higher age-group (above 35), the proportion of modern contraceptive users declined between baseline and endline. This might be because mostly women in higher age groups have no more desire for other children at baseline. Likewise, a decrease in the number of modern contraceptive users was seen over time among highly educated women, which might be due to the fact that most of them have completed their fertility desire and have opted for sterilization, which is not considered in this analysis.

It is worth noting that shift from a desire for more children to no more children is higher in proportion than the shift in the opposite direction. Since there is almost five-year gap between baseline to endline, many couples will have completed their family size during this interval, thus having no more desire for another children. Our hypothesis that women who desired more children at baseline would report lesser modern contraceptive use at endline compared to baseline was true for all cities except Aligarh, but did not attain significance in Agra and Varanasi. Agra reported the lowest (4.6%) and Aligarh the highest (10.4%) growth in modern contraceptive users over the period between baseline to endline ^28, 29^. The comprehensive increase in modern contraception at Aligarh between baseline and endline comprises an increase in modern contraceptive use not only in women who desired no more children at baseline but also for women who desired more children at baseline. These findings suggest a discrepancy between reported fertility intentions and subsequent contraceptive behavior in the Aligarh cohort. Another possible reason for this result might be the successful implementation of the “Happy Dampatti” contest, conducted by Johns Hopkins Bloomberg School of Public Health Center for Communication Programs with the help of government officials, NGOs and media, which reached 1.21 million people ^36^. As we hypothesized, women who did not desire children anymore at baseline reported greater contraceptive use at endline compared to baseline, which was true in all the cities except Agra (when adjusted for confounders), but significance was not reached in Agra and Varanasi. However, the reasons for these unexpected findings in Agra is not clear; it is possible that the swap of the effect noted in Agra might be due to some confounders that were not considered here. In India, generally women marry at an early age, and after having 2–4 children choose to be sterilized. These women may not expect to conceive soon; on the contrary, they may not want to avoid having a child until they become sterilized. This might create equivocal feelings in her to use of modern contraception. Previous studies ^11, 14, 32^ illustrated that women with equivocal feelings about future pregnancy at follow up were less likely to use contraceptives and had higher chance of reporting irregularity in use of contraception compared with women with a strong desire to avoid pregnancy.

A longitudinal study from Bali, Indonesia ^37^ concluded somewhat distinct results that “ *ambivalent women were not at high risk of unwanted pregnancies, indicating that they probably do not have a strong unmet need family planning services*”, ** and recommended that Demographic Health Survey (DHS) should exclude ambivalent women from the definition of unmet need.

### Strengths and limitations

If fertility preferences precisely predict subsequent contraceptive and fertility behavior, exploring fertility intentions can put forward policies that may be effective in helping women to meet their reproductive aspirations. Targeting women with equivocal feelings about fertility desires may be ineffective. Therefore, it is imperative to recognize and distinguish women who are less likely to use FP methods for effectively formulation of working strategies for targeting FP programs. Our results can help policy framers to set their goals. If the women who do not desire to resume childbearing and unaware of being at risk of unwanted pregnancy, delivering FP services and information could be a fruitful strategy to lessen the burden of unmet need along with maternal mortality and unintended pregnancies. The other significance of this study is that we have used longitudinal data to study the relationship between fertility desires and contraceptive use. Since fertility desires and contraceptive behavior changes over time, it is better to use longitudinal data instead of cross-sectional data to explain the relationship between the two. However, several limitations should be acknowledged here. First, our study did not specify the time period for the desire for another child (like the 2 or 3 years frequently used in other studies). Like other longitudinal studies, our study also suffers from attrition in the sample over time. Another limitation that needs to be address that we have assessed only women’s fertility intention instead of couples’ fertility intentions, since the proportion of couples who differed in their fertility desires was less than one percent. 

## Conclusions

At all six cities considered for effectiveness of Urban Health Initiative in Uttar Pradesh, women’s fertility intentions have an impact on their use of modern contraceptives over the time period between baseline to endline. Women with a desire for no more children reported higher use of modern contraception than women who desire more children at both baseline and endline. In Allahabad, Gorakhpur and Moradabad, women with no desire for another child at baseline reported lesser use of modern contraceptives at endline compared to baseline, while in Aligarh, Allahabad, Gorakhpur and Moradabad, women who desired more children at baseline reported higher use of modern contraceptives at endline compared with baseline. Our results differed across cities, showing that city-specific variations matter. To obtain greater insight into city-level differences, mixed method studies can be useful. It is worthwhile to mention that current family planning programs must recognize the dynamics of fertility intention and demand for modern contraception so that service providers can offer most suitable contraceptives to women that need it, instead of targeting women with ambivalent fertility motivations.

## Data availability

The underlying data for this study are taken from the Measurement, Learning & Evaluation Project of the Urban Reproductive Health Initiative. Ethical concerns surrounding the identifiability of responses means that access to these data is restricted. However, readers and reviewers can apply for access to the data by reading and completing the Data Use Agreement, providing intent of use, and returning it to the MLE Data Manager at the Carolina Population Center (email: mle-data-use-inquiry@unc.edu). 
