# Supplementary material for: The role of change in fertility desire on change in family planning use: A longitudinal investigation in urban Uttar Pradesh, India
Source: Gates Open Res. 2019 Jun 18;3:1439. Originally published 2019 Apr 29. [Version 2] doi: 10.12688/gatesopenres.12956.2 (PMC6545522; doi:10.12688/gatesopenres.12956.2)
Supplement: Peer review history

# Peer review: Reviewer response for version 1

**DOI:** 10.21956/gatesopenres.14062.r27097

**Published:** 2019-05-30

**Authors:** Tapan Kumar Roy

**Affiliations:** 1 Department of Population Science and Human Resource Development, University of Rajshahi, Rajshahi, Bangladesh

This paper has special scientific merit in the field of Fertility and Family Planning in India. It also plays an important contribution in reproductive health in India. Fertility desires and contraceptive use are strongly related in Asian over populated countries. In reproductive rights, women have the freedom to choose any methods but women are ambivalent towards desires of fertility and choice of contraception. In this regard, authors observed the change in fertility desires impact on use of modern contraception over time in six cities of Uttar Pradesh. They used data from the Measurement, Learning and Evaluation Project for Urban Health Initiative in six cities of Uttar Pradesh. This is panel data and authors handled properly in excellent to estimate the impact of change in modern contraceptive use over time among women who desired more children compared to women who desired no more children at baseline. Propensity score have been used equalizing women who desired no more and women who desired more children at baseline with matching to know similar distribution of observed baseline covariates. Again, inverse probability weighing is used to adjust selection bias due to loss to follow-up. Afterwards, authors have been employed generalized linear model (binary logistic regression: GLM with binomial family with logit link function) to find out the effect of confounding variables and time. However, the introduction and reference section may be updated with recent and updated literatures.

 Authors observed the factors affecting modern contraceptive use as well as fertility intention of six cities. It has also been investigated the city specific change of fertility desires and change in modern contraceptive use between baseline to endline. It is worthwhile to mention that the tempo of change in modern contraception over time among women with different fertility desires differed across the cities. This study will be a milestone for family planning policy makers to recognize the dynamics of fertility intention and demand for modern contraceptive users who are actually needed.

 Finally, I strongly recommend accepting the article.

I confirm that I have read this submission and believe that I have an appropriate level of expertise to confirm that it is of an acceptable scientific standard.

# Response

**Published:** 2019-06-05

**Authors:** Ujjaval Srivastava

We would like to thank you for taking the time and effort necessary to review the manuscript. We sincerely appreciate all valuable comments and constructive remarks, which helped us to improve the quality of the manuscript.

# Peer review: Reviewer response for version 1

**DOI:** 10.21956/gatesopenres.14062.r27143

**Published:** 2019-05-14

**Authors:** Sada N. Dwivedi

**Affiliations:** 1 Department of Biostatistics, All India Institute of Medical Sciences (AIIMS), New Delhi, Delhi, India

From reproductive health programme point of view, this article is very much relevant, more so because its inferences rely on data of a large sample. However, following points may help in further strengthening of the article:

1. Under research hypotheses, there is need to differentiate between research question and related hypothesis. It would be appreciated, if three research questions are written first, and corresponding research hypothesis may be listed later. As a convention, they need to be written using alternative hypotheses.
2. Further, respective objectives of the study may be listed.
3. It is not clear that why modern contraceptives  do not include  practice of safe period method. These may be spelled out clearly in the article.
4. How were the baseline education, parity, wealth, caste, religion, age, residence and having a son at home selected as potential confounders? What about others like gender of last child, living status of last child?
5. Was the collinearity  among the covariates/ confounders explored?
6. Further, was the first order effect modifiers for the role of fertility desire on change in family planning use explored.
7. How would one interpret the lower shift from non-users at baseline to users at end-line in comparison to higher shift from modern contraceptive users  at baseline to non-users at end-line (Table 4)?
8. In my opinion, keeping in view of city specific analytical results, analysis for pooled data may also be carried out and included, If required, to take into account city-level variability, city may be included as a co-variate in this regard.

Hope consideration of these points may help in further strengthening of the article.

I confirm that I have read this submission and believe that I have an appropriate level of expertise to confirm that it is of an acceptable scientific standard.

## References

[1] : Epidemiological models and related simulation results for understanding of contraceptive adoption in India. *Int J Epidemiol*.2000;29(2) :300-7 1081712910.1093/ije/29.2.300

# Response

**Published:** 2019-05-20

**Authors:** Ujjaval Srivastava

Many thanks to the reviewer for their positive feedback and constructive suggestions, which we appreciate. I feel that readers will likely find our paper new and interesting. Our response follows:

**Reviewer’s comment (1):** Under research hypotheses, there is need to differentiate between research question and related hypothesis. It would be appreciated, if three research questions are written first, and corresponding research hypothesis may be listed later. As a convention, they need to be written using alternative hypotheses.

**Reply:** Thank you for highlighting this mistake. I should use only “research hypothesis” instead of research question here because for getting the answer to our research question, I have converted it into testable research hypothesis statement. I will correct it into revised version of manuscript.

**Reviewer’s comment (2):** Further, respective objectives of the study may be listed.

**Reply:** The objective of our study is to explore the relationship between women’s fertility desires and their contraceptive behaviour through causal inference in urban settings of Uttar Pradesh, India and broad objectives are given in terms of research hypothesises.

**Reviewer’s comment (3):** It is not clear that why modern contraceptives do not include practice of safe period method. These may be spelled out clearly in the article.

**Reply:** In our data set, the frequency of ‘Standard days method’ is zero. That is why we do not mention about it in the article.

**Reviewer’s comment (4):** How were the baseline education, parity, wealth, caste, religion, age, residence and having a son at home selected as potential confounders? What about others like the gender of last child, the living status of last child?

**Reply:** I have clearly mentioned the procedure to select potential confounding variables in statistical analysis section. Though I have not checked for gender of last child, living status of last child as confounding variable but I appreciate your suggestion and consider these into my future work.

**Reviewer’s comment (5):** Was the collinearity among the covariates/ confounders explored?

**Reply:** Yes, we have tested for the collinearity among the confounders and we found that there was no significant multicollinearity exist.

**Reviewer’s comment (6):** Further, was the first order effect modifiers for the role of fertility desire on change in family planning use explored.

**Reply:** No, we have not explored the first order modifiers in this problem.

**Reviewer’s comment (7):** How would one interpret the lower shift from non-users at baseline to users at end-line in comparison to higher shift from modern contraceptive users at baseline to non-users at end-line (Table 4)?

**Reply:** I have not written this interpretation in anywhere in the manuscript. However, in discussion section, I have written following sentence “ *It is worth noting that shift from a desire for more children to no more children is higher in proportion than the shift in the opposite direction*” regarding the change in fertility desires.

**Reviewer’s comment (8):** In my opinion, keeping in view of city specific analytical results, analysis for pooled data may also be carried out and included, If required, to take into account city-level variability, city may be included as a co-variate in this regard.

**Reply: **I have also carried out whole analysis on pooled data with city as an additional covariate but we want to test our hypothesizes in each city. Due to limitation of length of the research paper, we have not shown the pooled results and showed only city wise results.
